# Brain tumor classification using MRI images and deep learning techniques

**DOI:** 10.1371/journal.pone.0322624

**Published:** 2025-05-09

**Authors:** Yuki Wong, Eileen Lee Ming Su, Che Fai Yeong, William Holderbaum, Chenguang Yang

**Affiliations:** 1 Faculty of Electrical Engineering, Universiti Teknologi Malaysia, Johor Bahru, Malaysia; 2 Manchester Metropolitan University, Manchester, United Kingdom; 3 University of Liverpool, Liverpool, United Kingdom; University of Sargodha, PAKISTAN

## Abstract

Brain tumors pose a significant medical challenge, necessitating early detection and precise classification for effective treatment. This study aims to address this challenge by introducing an automated brain tumor classification system that utilizes deep learning (DL) and Magnetic Resonance Imaging (MRI) images. The main purpose of this research is to develop a model that can accurately detect and classify different types of brain tumors, including glioma, meningioma, pituitary tumors, and normal brain scans. A convolutional neural network (CNN) architecture with pretrained VGG16 as the base model is employed, and diverse public datasets are utilized to ensure comprehensive representation. Data augmentation techniques are employed to enhance the training dataset, resulting in a total of 17,136 brain MRI images across the four classes. The accuracy of this model was 99.24%, a higher accuracy than other similar works, demonstrating its potential clinical utility. This higher accuracy was achieved mainly due to the utilization of a large and diverse dataset, the improvement of network configuration, the application of a fine-tuning strategy to adjust pretrained weights, and the implementation of data augmentation techniques in enhancing classification performance for brain tumor detection. In addition, a web application was developed by leveraging HTML and Dash components to enhance usability, allowing for easy image upload and tumor prediction. By harnessing artificial intelligence (AI), the developed system addresses the need to reduce human error and enhance diagnostic accuracy. The proposed approach provides an efficient and reliable solution for brain tumor classification, facilitating early diagnosis and enabling timely medical interventions. This work signifies a potential advancement in brain tumor classification, promising improved patient care and outcomes.

## Introduction

The classification of brain tumors from Magnetic Resonance Imaging (MRI) images using deep learning (DL) techniques is a crucial area of research in the medical field. The brain is a complex and extremely sensitive organ of the human body [1]. Brain tumors are abnormal growths of cells when the behavior of morphological cells in the brain is influenced by inappropriate mitosis procedures that can have severe consequences on the health and well-being of individuals [2]. The World Health Organization (WHO) classifies brain tumors into different grades, with low-grade tumors being slow-growing and benign and high-grade tumors being more aggressive and malignant [3]. According to statistics approved by Cancer.Net Editorial Board, brain tumors are the 10^th^ leading cause of cancer-related death [4]. The most prevalent brain tumor types in adults are meningioma and glioma, which make up 81% of malignant brain tumors in adults [5]. Therefore, early detection and accurate classification of brain tumors are essential for effective treatment and improving patient outcomes.

Brain tumor diagnosis relies heavily on visual inspection by radiologists, which can be time-consuming, tedious, and prone to human error. This is because most cells typically consist of a portion of arbitrary, random, and uncontrolled perspectives [6]. Since traditional diagnostic techniques have limitations in tumor detection, there is a need for computer-aided methods with higher accuracy. Recent advancements in artificial Intelligence (AI) and DL, particularly convolutional neural networks (CNNs), have demonstrated immense potential in medical imaging analysis. Studies, such as Liu et al.’s comparison of DL models and healthcare professionals, suggest that DL systems can achieve diagnostic performance equivalent to expert radiologists [7], further underscoring their relevance in tumor detection and classification. Besides, MRI is widely regarded as the preferred modality for brain tumor detection and classification due to its ability to provide detailed information and analyze tumor regions from different angles [8]. Within this context, CNNs are especially effective, as their built-in convolutional layers excel at reducing the high dimensionality of MRI images while retaining essential information for accurate analysis [9].

This study aims to advance brain tumor diagnosis by developing a robust CNN-based model to classify brain MRI images into four categories: glioma, meningioma, pituitary tumors, and normal scans. Moving beyond binary classification focus of prior studies, this research takes a multiclass approach, offering a more comprehensive solution for tumor diagnosis. The proposed model leverages the pretrained VGG16 architecture, renowned for its exceptional feature extraction capabilities. By integrating additional layers and applying fine-tuning techniques, the model is optimized to improve feature recognition and classification accuracy. To address limitations of small and less diverse datasets commonly seen in previous research, this study combines three large public datasets and employs data augmentation techniques, increasing the dataset size from 5,712–17,136 images. This enhanced dataset ensures a balanced and comprehensive representation of tumor classes, enabling the model to achieve high accuracy across diverse cases. Moreover, the study bridges research and practical application by developing a user-friendly web application with a graphical user interface (GUI). This tool allows for seamless MRI image uploads and real-time tumor predictions, making it accessible for healthcare professionals or researchers.

In summary, this study makes significant contributions, including combining large public datasets to address dataset limitations, applying data augmentation to enhance model training, expanding classification to four tumor types, optimizing CNN architecture for improved performance, and developing a practical application for real-world use. Achieving an impressive accuracy of 99.24%, this system demonstrates the potential to improve diagnostic precision and efficiency, representing a valuable advancement in DL-based medical imaging and brain tumor diagnosis.

### Research contributions

- Developed an automated brain tumor classification system using DL and MRI images.
- Employed a pretrained VGG16 CNN architecture, achieving an accuracy of 99.24%, higher than comparable models.
- Utilized a large and diverse dataset with 17,136 brain MRI images, including glioma, meningioma, pituitary tumors, and normal brain scans.
- Implemented data augmentation and fine-tuning strategies to enhance classification performance.
- Created a user-friendly web application for easy image upload and real-time tumor prediction.
- Demonstrated the potential of the system to reduce human error and improve diagnostic accuracy in clinical settings.

## Related works

The application of DL techniques, particularly CNNs, in the classification of brain tumors using MRI images has been extensively studied. This section reviews recent advances in this area and highlights the limitations of existing approaches while presenting the motivation for the proposed method.

Several studies have explored the application of CNN models in brain tumor classification, utilizing various techniques and architectures. H. Khan et al. [8] employed CNN models such as VGG-16, ResNet-50, and Inception-v3, along with data augmentation and image processing techniques. Their study achieved an impressive 100% accuracy, highlighting the effectiveness of CNN models in this context. However, the study was limited by its focus on binary classification and the utilization of a small dataset, which may affect the generalizability of the results. Similarly, S. Das et al. [10] adopted a CNN approach for brain tumor classification, employing preprocessing techniques on MRI images. Their dataset consisted of three types of brain tumors: glioma, meningioma, and pituitary. The CNN approach achieved a testing accuracy of 94.39%, with high precision and recall. However, the study was constrained by the limited dataset size and lacked the diversity needed for robust model performance. Meanwhile, S. Pokhrel et al. [11] explored various CNN models, including MobileNetV2, MobileNetV3-small, MobileNetV3-large, VGG16, VGG19, and a custom CNN model. They focused on distinguishing between normal and abnormal pixels and classifying tumor-affected brains using real-world datasets. The results indicated high accuracy rates, with different CNN models achieving accuracies ranging from 94.17% to 97.17%. However, the absence of data augmentation techniques and the focus on binary classification present opportunities for further improvement and exploration in this area.

In an alternative approach, T. Hossain et al. [12] combined the fuzzy C-means clustering algorithm with traditional classifiers and CNN models. Their study achieved an accuracy of 97.87% with CNN, showcasing its potential in brain tumor classification. However, similar to other studies, the research gap lies in the utilization of a small dataset and the focus on binary classification, which may limit its broader applicability. P. Gokila Brindha et al. [13] compared artificial neural network (ANN) and CNN models for brain tumor detection. The CNN model outperformed the ANN, achieving higher accuracy, F1 score, and precision. However, the study suffered from a small amount of image data and the lack of optimization for the trained network. This suggests avenues for future research to improve the dataset size and optimize the network’s performance. Another notable study by M. Abu et al. [14] employed a deep convolutional neural network (DCNN) for brain tumor classification using MRI images. They utilized a pretrained VGG-16 model through transfer learning and implemented data augmentation techniques. The results demonstrated high accuracy, precision, recall, F1-score, Cohen’s Kappa, and AUC. Notably, the proposed methodology did not require extensive training, which is a significant advantage. However, the study was limited by a very small dataset and focused solely on binary classification.

Furthermore, Pillai et al. [15] utilized transfer learning models, including VGG16, ResNet50, and Inception V3, for brain tumor classification on a dataset of 251 MRI scans. They enhanced model performance by adding fine-tuning layers such as Dropout, Flatten, and Dense to the pretrained models. The results indicated that VGG16 achieved a training accuracy of 97.24% and a validation accuracy of 91.58%. InceptionV3 and ResNet50 achieved lower validation accuracies of 63.86% and 81.94%, respectively. However, the study primarily focused on binary classification and employed a relatively small dataset, which may limit the generalizability of the findings. In addition, existing studies have claimed that higher accuracy can be achieved by using smaller datasets [16]. However, the use of smaller datasets may result in the exclusion of certain images, especially if they do not yield satisfactory results. Consequently, when applied to larger and noisy datasets, the practical implementation of the brain tumor classification system for medical purposes may not attain the same level of accuracy.

The main limitations observed across these studies include the use of small datasets, which hampers generalizability, and a focus on binary classification, which restricts the models’ ability to distinguish between multiple tumor types. Furthermore, few studies incorporate robust data augmentation techniques, and there is a notable absence of user-friendly interfaces to facilitate practical clinical applications. In contrast, this study addresses these limitations by utilizing a significantly larger and more diverse dataset, consisting of 17,136 MRI images across four tumor classes (glioma, meningioma, pituitary, and normal brain). The inclusion of data augmentation techniques further enhances the training process by increasing the dataset’s size and diversity. Additionally, a fine-tuning strategy is applied to a CNN model with a pretrained VGG-16 base, which enables the network to leverage the advantages of transfer learning while optimizing it for brain tumor classification. Unlike previous studies, this work also develops a web-based application to make the system more accessible to healthcare professionals, allowing for easy image upload and tumor prediction.

In this study, the glioma, meningioma, and pituitary tumors are chosen for investigation due to their mortality and severity rate at current times. To diagnose these diseases, MRI imaging is comparatively economical, fast, painless, and suitable for brain tumor diagnosis. Hence, many researchers chose MRI as the type of input image for their brain tumor detection and classification system. Based on the literature review, CNN was selected as the architecture for this project due to its superior performance in other research. Data augmentation was employed to increase training data diversity, effectively addressing overfitting and improving model generalization.

Table 1 presents a comparative analysis of the related work in this domain, highlighting the key aspects of each study.

**Table 1:** Comparative analysis of related work in brain tumor classification using CNNs.

Authors (Year)	Architecture	Dataset Size	Tumor Classes	Accuracy	Limitations
H. Khan et al. (2020) [8]	VGG-16, ResNet-50, Inception-v3	253 brain MRI images	2 (Glioma, Meningioma)	CNN: 100% VGG-16: 96% ResNet-50: 89% Inception-V3: 75%	Small dataset Binary classification Limited generalizability
S. Das et al. (2019) [10]	CNN	3064 brain MRI images	3 (Glioma, Meningioma, Pituitary)	94.39%	Small dataset Limited to three tumor types
S. Pokhrel et al. (2022) [11]	MobileNetV2, MobileNetV3-small, MobileNetV3-large, VGG16, VGG19 and CNN	3000 brain MRI images	2 (Tumor, Non-tumor)	MobileNetV2: 94.17% MobileNetV3-small: 94.83% MobileNetV3-large: 94.83% VGG16: 97% VGG19: 97% CNN: 97.17%.	Small dataset No data augmentation Binary classification
T. Hossain et al. (2019) [12]	Traditional classifiers, CNN with Fuzzy C-Means Clustering	217 brain MRI images	2 (Tumor, Non-tumor)	SVM: 92.42% CNN: 97.87%	Small dataset Binary classification
P. Gokila Brindha et al. (2021) [13]	CNN, ANN	2065 brain MRI images	2 (Tumor, Non-tumor)	ANN: 65.21% CNN: 89%	Small dataset No network optimization Binary classification
M. Abu et al. (2020) [14]	DCNN based on pretrained VGG-16 model	253 brain MRI images	2 (Tumor, Non-tumor)	96%	Small dataset Binary classification
Pillai et al. (2023) [15]	VGG16, ResNet50, Inception V3 transfer learning models with fine-tuning layers	251 brain MRI images	2 (Tumor, Non-tumor)	VGG16: 91.58% ResNet50: 81.94% InceptionV3: 63.86%	Focused on binary classification Relatively small dataset
**This work**	**CNN (VGG16 base model with data augmentation)**	**17,136 brain MRI images**	**4 (Glioma, Meningioma, Pituitary, Normal)**	**99.24%**	**–**

## Methods

This section provides a detailed account of the methodology, encompassing data acquisition, preprocessing, architectural design, and performance evaluation of the deep learning model. Advanced computational techniques, including transfer learning and fine-tuning, are employed to maximize the utility of existing neural networks while customizing them for brain tumor classification. The study is structured into three primary stages: training and validation, testing, and implementation, supported by rigorous data preprocessing, model optimization, and evaluation strategies to ensure robust performance. A custom dataset, constructed from multiple public sources, underpins the model’s development, and the approach is further enabled by specific tools and hardware configurations for efficient execution.

### Process overview

**Fig 1:**
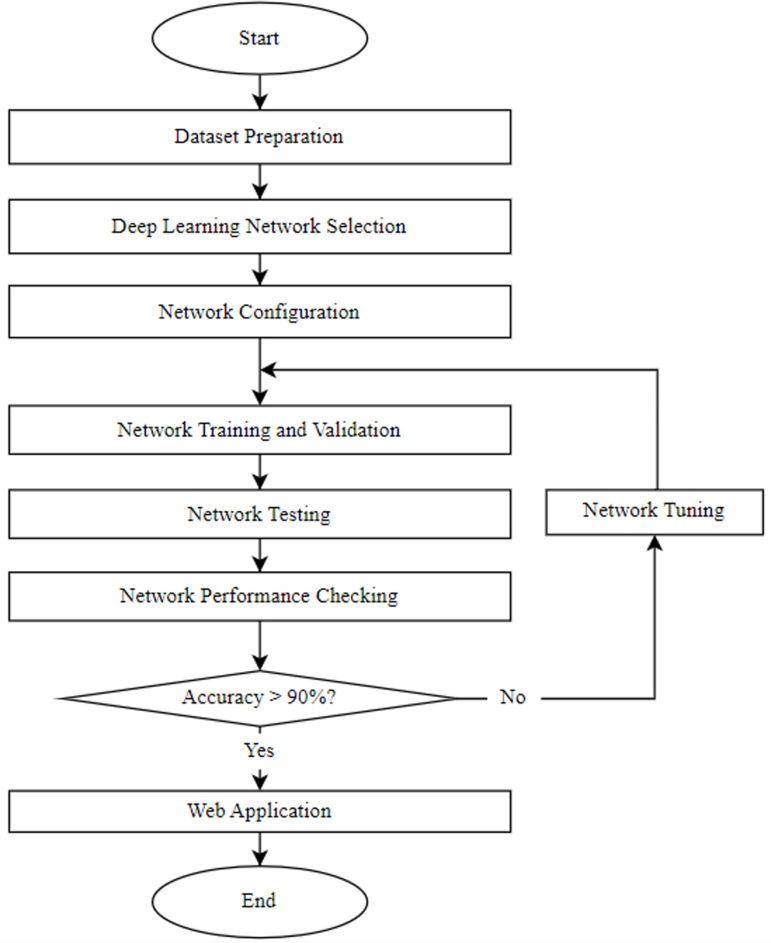
Flowchart of the proposed methodology.

This research comprised three primary phases: training and validation, testing, and implementation of the brain tumor classification model. Fig 1 provides a detailed flowchart outlining the fundamental steps of the proposed methodology.

First, all necessary libraries were identified and imported, as shown in Fig 1. These libraries included Keras, numpy, matplotlib, sklearn, and os. Following the import of libraries, the dataset was prepared, organizing training images, validation images, and testing images. The dataset was then split into training, validation, and testing sets in the ratio of 8:1:1. A pretrained VGG16 model was employed as the base model, with all its layers frozen to retain pre-learned features. The DL model was then compiled, trained, evaluated, and implemented. If the model’s accuracy did not exceed 90%, the number of epochs was adjusted, and the model was retrained to enhance performance. Once the model was fully trained, a web application was developed to predict and classify different types of brain tumors.

### Development tools

The development tools used for model training and testing in this research project included the Python programming language. The laptop was equipped with an Intel Core i5-1135G7 processor with a base frequency of 2.40 GHz. The graphics were handled by Intel(R) Iris(R) Xe Graphics. The laptop had 8 GB of memory and 476 GB of storage. The operating system used was Windows 11. These hardware specifications were utilized to carry out the necessary computations and run the Python-based development environment for training and testing the models.

### Dataset

The dataset used in this study for brain tumor classification was collected by combining multiple selected public MRI datasets, as shown in Table 2. Relevant datasets from various sources were sought to gather a comprehensive collection of brain tumor images.

**Table 2:** Selected MRI datasets for training, validation, and testing.

Classes	Dataset	Number of Images
Pituitary Tumor	Figshare [17] SARTAJ dataset [18]	856 901
Glioma Tumor	Figshare [17]	1621
Meningioma Tumor	SARTAJ dataset [18] Figshare [17]	937 708
No Tumor	Br35H [19] SARTAJ dataset [18]	1500 500

The data collection process involved utilizing three primary datasets. The first dataset was obtained from Figshare, specifically “Version 5” posted on April 3, 2017 [17]. This dataset comprised 3064 T1-weighted contrast-enhanced images featuring three different types of brain tumors. The second dataset, known as the SARTAJ dataset, contributed a portion of the data. The “no tumor” class data were originally sourced from the Kaggle dataset [18]. To ensure data quality, Sartaj Bhuvaji and his team conducted a manual examination of the images. They identified certain images in the “no tumor” class that exhibited visible tumors. These images were subsequently removed from the dataset to maintain accuracy. To further validate the dataset, they sought the expertise of a doctor who thoroughly examined all the MRIs and provided a certificate supporting the project’s objectives [18]. In addition, the SARTAJ dataset had a problem in that the “glioma tumor” class images were not categorized correctly [20]; thus, the “glioma tumor” class images on the Figshare site were used instead of the images from SARTAJ. The third dataset utilized in this study was the Br35H dataset, specifically the “Brain Tumor Detection 2020” collection. The images from this dataset were incorporated to augment their custom dataset, specifically for the “no tumor” class.

By amalgamating the data from these sources, a custom dataset was created, comprising brain tumor images categorized into four different classes: pituitary tumor, glioma tumor, meningioma tumor, and no tumor. The dataset contained a significant number of images, with the “pituitary tumor”, “glioma tumor”, “meningioma tumor”, and “no tumor” classes consisting of 1757, 1621, 1645, and 2000 images, respectively. The overall data collection process involved sourcing publicly available MRI datasets. This carefully curated custom dataset served as the foundation for training and evaluating the DL model utilized in the study. Figshare, SARTAJ, and Br35H datasets were combined in an approximate ratio of 4:3:2. The number of images for each tumor or no tumor category from each of the datasets is shown in Table 2. The combined dataset is then split based on class into an 8:1:1 ratio for training, validation, and testing processes.

### Preprocessing

Preprocessing played a crucial role in preparing the dataset for training a DL model. It involved a series of steps that were designed to enhance the performance and generalization ability of the model. In this study, several preprocessing techniques were applied to the brain tumor dataset, aiming to optimize the training process and improve the model’s accuracy.

The grayscale conversion step ensured that the images were represented in a single channel, containing only intensity information. By converting the images to grayscale, color information was discarded, simplifying the data and reducing computational complexity. This simplification is suitable for brain MRI images, where the intensity variations hold crucial information for analysis and classification.

To increase the diversity of the training data and improve the model’s ability to generalize to unseen examples, data augmentation techniques were applied. These techniques introduce slight modifications to the original images, generating additional training samples with variations. In this study, the data augmentation techniques employed included rescaling, shearing, zooming, and horizontal flipping. Each augmentation parameter served a specific purpose:

1. **Rescaling**: The rescaling parameter normalized the pixel values by dividing them by 255. This normalization step ensures that the pixel values fall within the range of 0–1 and aids in the convergence of the training process. Normalizing the data to smaller values is a common practice in DL to enable the model to learn more effectively and speed up convergence.
2. **Shear Range**: Augmentation with a range of shear transformations introduced variations in the orientation of the images. This technique helps the model become more resilient to different angles and geometric distortions that may occur in brain MRI images, improving its ability to accurately recognize and classify structures.
3. **Zoom Range**: Applying a range of zoom values during augmentation simulated variations in the scale or magnification of the images. By training on images at different levels of magnification, the model learns to handle images with varying sizes and zoom levels, enhancing its ability to generalize and classify unseen images effectively.
4. **Horizontal Flip**: The horizontal flip augmentation horizontally mirrored the images. This transformation introduces variations that occur naturally, such as differences in orientation or anatomical structures from left to right. By training on both the original and flipped versions of the images, the model becomes more robust to these variations.

The augmentation techniques were specifically applied to the training set, while the validation and test sets remained unaltered. This separation ensures that the model’s performance is evaluated on the original, unaltered data, providing a reliable assessment of its generalization ability. The data augmentation and normalization steps were implemented using the Keras library, which provides convenient tools for image preprocessing. The “ImageDataGenerator” class in Keras allows on-the-fly data augmentation during the training process. It generates batches of augmented images based on the specified augmentation techniques and batch size. To efficiently generate augmented image batches during training, the “flow_from_directory” method from the Keras library was utilized. This method reads and augments images from the directory structure, reducing memory consumption and enabling the training of models with large datasets. By incorporating these augmented images into the training dataset, the model becomes more robust to variations in the test data, leading to improved generalization and performance.

Furthermore, concatenation was implemented in the preprocessing to concatenate the original and augmented images for the training data. A custom function called “concatenate_images” was implemented to iterate through each image in the training directory, apply augmentation transformations, and save the augmented images. For each original image, two augmented images were generated using the data generator. This augmentation strategy resulted in a threefold enhancement of the dataset size, increasing it from 5,712–17,136 samples. This expanded dataset not only provided a larger pool of training examples but also introduced greater diversity in tumor patterns and variations, thereby enhancing the model’s exposure to various scenarios and improving its ability to generalize to unseen data.

Besides, the number of images in the training, validation, and testing directories was counted. This count provides an overview of the dataset distribution, enabling researchers to assess the balance of the dataset and identify any potential data imbalances or biases that might affect the model’s training and evaluation. To prevent overfitting and to ensure reliable evaluation of the model, the dataset was split into training, validation, and testing sets. An 8:1:1 ratio was employed, where 80% of the data were allocated for training and the remaining 20% were evenly divided between the validation and testing sets. This partitioning helps assess the model’s performance on unseen data and provides a basis for adjusting hyperparameters and fine-tuning the model.

The preprocessing steps applied in this study aimed to optimize the brain tumor dataset for DL model training. The model’s performance and generalization ability were enhanced by organizing the dataset, splitting it into appropriate sets, applying data augmentation techniques, and normalizing the data. The augmentation diversified the training data, making the model more robust to variations in the test data. The normalization ensured consistent data representation, and the dataset organization facilitated efficient data management. These preprocessing steps collectively contributed to improved model accuracy and reliability in brain tumor detection and classification tasks. The generated dataset is explained in further detail in the Results section of this paper.

### Deep learning architecture

The chosen DL architecture for the brain tumor classification model was a CNN with a pretrained VGG16 model as the base model. The VGG16 model is a popular CNN architecture that has demonstrated strong performance on various computer vision tasks.

The network structure consisted of the following:

1. Pretrained VGG16 Model: This model included an Input Layer that accepted input images with dimensions of (224, 224, 3). It comprised 13 convolutional layers organized into five blocks, with each block followed by a max-pooling layer for down-sampling. This architecture effectively learned hierarchical representations of the input images, extracting relevant features for classification tasks.
2. Flatten Layer: This layer flattened the output from the previous layer into a 1D vector, preparing it for the subsequent dense layers.
3. Dense Layer (256 neurons, ReLU activation): This fully connected layer with 256 neurons used the rectified linear unit (ReLU) activation function to introduce nonlinearity and learn complex patterns and features.
4. Dense Layer (4 neurons, softmax activation): This final fully connected layer had 4 neurons, representing the four different classes of brain tumors. It used the softmax activation function to produce class probabilities for multiclass classification.

The model was trained using the Adam (Adaptive Moment Estimation) optimization algorithm. In the initial training phase, a learning rate of 0.001 was used, and in the subsequent training phase, a learning rate of 0.0001 was used. The Adam optimizer helped optimize the model’s weights and biases, improving its ability to accurately classify brain tumors.

### Modifications and fine-tuning

Transfer learning was applied using the pretrained VGG16 model. This strategy allows storing and leveraging the knowledge gained from the pretrained VGG16 model on a large-scale dataset and applying that knowledge to the brain tumor classification task [21]. All layers of the model, except the last two dense layers, were frozen or made nontrainable. This allowed the network to leverage the pretrained model’s learned features while adapting the last two layers specifically for the brain tumor classification task.

To perform a technique called fine-tuning [22], the model was trained twice. During the first stage of training, the nontrainable parameters from the VGG16 model remained fixed, while only the parameters in the last two dense layers were updated with a learning rate of 0.001. This fine-tuning strategy enabled the model to benefit from the knowledge gained from a large-scale dataset on which the VGG16 model was originally trained while specializing in its learning to the specific brain tumor classification problem.

After the initial training stage, the top few layers of the pretrained model were unfrozen to perform further fine-tuning. The first ten layers are set as nontrainable, while the remaining layers starting from the 11th layer are set as trainable. This selective unfreezing allows specific parts of the model to adapt to the new dataset. After unfreezing the layers, the entire model was retrained with a smaller learning rate of 0.0001. This allowed the unfrozen layers to adapt to the specific features of the new dataset while still retaining the knowledge from the pretrained layers. The lower learning rate would help in fine-tuning the newly unfrozen layers without causing significant changes to the already trained layers. By using this two-stage training approach with frozen and unfrozen layers, the fine-tuning strategy allows the model to learn more specialized features from the new dataset, improving its performance on the brain tumor classification task.

### Network training and evaluation

The model was trained using the training set with a batch size of 32 and the Adam optimizer. The training process involved feeding the augmented images generated from the training set through the CNN model and updating the trainable parameters using backpropagation. Both the first and second stages underwent 50 epochs of training with 179 steps per epoch and 21 validation steps. The number of epochs was adjusted as needed to improve the model’s performance.

The model’s performance was evaluated using various metrics, including the confusion matrix, sensitivity, specificity, and per-class accuracy. A confusion matrix is a type of performance metric commonly used to assess the performance of a classification model for which the true values are known. It is a table with four different combinations of predicted and actual values: true-positive (TP), false-positive (FP), true-negative (TN), and false-negative (FN) [23]. These variables are fundamental in evaluating the performance of a classification model and understanding the accuracy of its predictions with respect to positive and negative cases.

TP represents the number of instances that are correctly identified as positive by the model. This means that the predicted positive outcomes align with the actual positive labels. TN, on the other hand, indicates the number of instances correctly identified as negative by the model, where the predicted negatives match the actual negative labels. FN represents the instances that are incorrectly classified as negative by the model, meaning that the predicted negatives do not match the actual positive labels. Finally, FP refers to the instances that are incorrectly classified as positive, where the predicted positives do not match the actual negative labels.

The validation set, which was separated from the training set, was used to monitor the model’s performance during training. Early stopping was employed to halt the training process if the model’s performance did not improve for a certain number of epochs. Once the training was completed, the model’s performance was tested using a separate testing set for each class. The trained model was applied to the testing set, and the performance metrics were calculated based on the model’s predictions. The following are the scoring metrics employed in this study. These metrics are denoted by Equations (1–5) as follows:

Sensitivity or recall was the measure of how much the correct prediction was from all the positive classes. The higher the sensitivity was, the better the performance of the trained model.


$$Sensitivity=\frac{TP}{TP+FN}$$ (1)

Precision was the measure of how much the actual positive from all classes the model had predicted as positive. The higher the precision was, the better the performance of the trained model.


$$Precision=\frac{TP}{TP+FP}$$ (2)

Specificity was the measure of the ability of a classification model to correctly predict the negative cases. A high specificity would indicate that the model has a low rate of false positives and was effective at identifying negative cases.


$$Specificity=\frac{TN}{TN+FP}$$ (3)

The F1-score was also known as the F score or the F measure. The F1-score gave a measurement based on a balance between precision and recall. The higher the F1-score was, the better the performance of the trained model.


$$F\text{1}-score=\frac{\text{2}\times \left(\frac{TP}{TP+FP}\right)\times \left(\frac{TP}{TP+FN}\right)}{\left(\frac{TP}{TP+FP}\right)+\left(\frac{TP}{TP+FN}\right)}$$ (4)

Accuracy was the measure of the correct prediction out of all classes. The higher the accuracy was, the better the performance of the trained model.


$$Accuracy=\frac{TP+TN}{TP+TN+FP+FN}$$ (5)

## Results

This section presents the outcomes of the study, highlighting the effectiveness of the developed brain MRI image classification system. The results encompass detailed analyses of the dataset generation process, training and validation performance, classification accuracy, and the robustness of the model as evaluated on a separate test dataset. Key metrics such as precision, recall, F1-score, sensitivity, specificity, and overall accuracy are analyzed to underscore the system’s reliability in detecting and classifying brain tumor types. Visualizations, including sample image augmentations, plots of accuracy and loss, and a confusion matrix, further elucidate the model’s behaviour and predictive performance. In addition, the integration of the trained model into a web application is discussed, showcasing its practical applicability for real-world usage.

### Generated dataset

**Fig 2:**
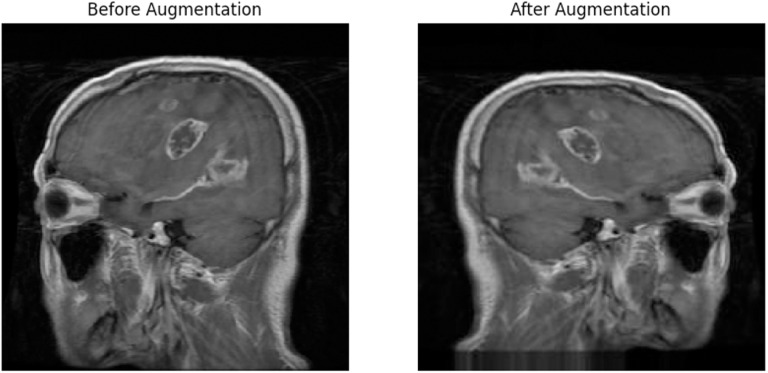
Sample images before and after augmentation.

The input image processing phase successfully transformed the dataset for training the model. The application of grayscale conversion and various augmentation techniques resulted in an expanded and diverse training dataset, which was essential for training a robust and accurate brain MRI image classification model. After the augmentation process, the training directory contained a total of 17,136 images belonging to four classes of brain MRI images. The validation and test directories also contained a substantial number of images, totaling 656 and 655, respectively, all belonging to the same four classes. Fig 2 illustrates sample MRI brain images before and after augmentation.

The input image processing techniques employed in this study successfully increased the variance and diversity of the training dataset. By incorporating grayscale conversion and applying augmentation parameters, the model was equipped to handle different variations, orientations, and scales commonly found in brain MRI images. These preprocessing steps were essential for training a model that can generalize well to unseen data and accurately classify different types of brain MRI images.

### Training and validation results

**Fig 3:**
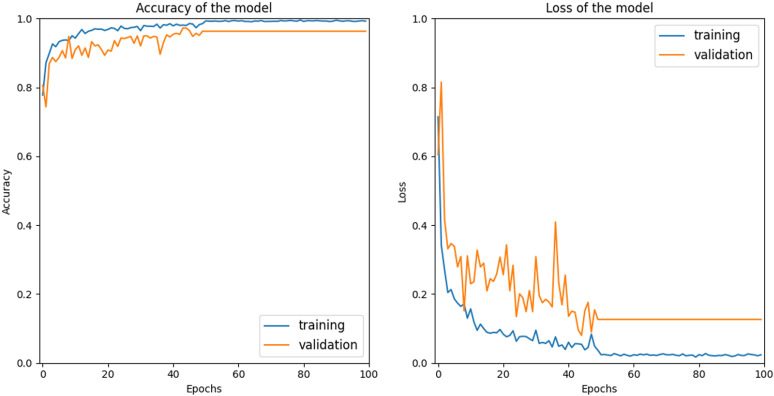
Accuracy and loss plots of the model.

The constructed network was compiled and trained using a total of 3,963 glioma MRI images, 4,017 meningioma MRI images, 4,371 pituitary MRI images, and 4,785 normal MRI images. The model’s performance was evaluated by feeding the validation data into the network and calculating the accuracy and loss. For validation, 150 glioma MRI images, 153 meningioma MRI images, 150 pituitary MRI images, and 203 normal MRI images were used. Fig 3 illustrates the accuracy and loss plots against the number of epochs.

The observed behavior, where the accuracy of the model increased as the number of epochs increased while the loss decreased, indicated a convergence of the model during the training process. Convergence referred to the point at which the model had learned the underlying patterns and relationships present in the training data, leading to improved performance.

As the model underwent training over multiple epochs, it progressively adjusted its parameters to minimize the loss function. The loss function quantified the discrepancy between the model’s predicted outputs and the actual labels. By iteratively updating the parameters based on the computed gradients, the model aimed to find the optimal set of parameters that minimize prediction errors. The increase in accuracy signified that the model’s predictions align more closely with the true labels as the training progresses. The model became better at correctly classifying or predicting the target variable, reflecting an improved understanding of the underlying patterns in the data.

Simultaneously, the decrease in loss indicated that the model was reducing its prediction errors. As the model updated its parameters to minimize the loss function, it gradually aligned its predictions with the actual labels, resulting in a lower overall loss. This decrease in loss demonstrated that the model was converging toward a state where its predictions were more accurate. However, it is important to consider the potential for overfitting during the convergence process. Overfitting could occur when the model becomes too specific to the training data and fails to generalize well to unseen data [24]. To mitigate overfitting, it was common to monitor the performance of the model on a separate validation set. If the accuracy on the validation set started to decrease or plateau while the training accuracy continued to improve, it may indicate that the model was overfitting.The observed convergence of the model, with increasing accuracy and decreasing loss over the course of training, suggested that the model was learning and improving its predictions. Careful monitoring and evaluation of the model’s performance on both training and validation data helped ensure that the convergence was indicative of generalization and not overfitting.

### Network performance

The trained model was assessed using a separate test dataset consisting of 150 glioma MRI images, 153 meningioma MRI images, 150 pituitary MRI images, and 202 normal MRI images. The “classification_report” function was employed to compute precision, recall, and F1-score for each class, as shown in Table 3.

**Table 3:** Precision, recall, and F1-score for each class.

	Precision	Recall	F1-score
**Glioma tumor**	0.99	1.00	1.00
**Meningioma tumor**	0.99	0.97	0.98
**No tumor**	1.00	1.00	1.00
**Pituitary tumor**	0.98	0.99	0.99

Based on the data in Table 3, the model demonstrated high performance in classifying different types of brain tumors. For glioma tumors, the precision was 0.99, the recall was 1.00, and the F1-score was 1.00. This indicates that the model was highly effective in identifying glioma tumors, with very few false positives, and correctly identified all true glioma cases. The “meningioma tumor” class showed a precision of 0.99, a recall of 0.97, and an F1-score of 0.98. While the precision was high, indicating few false positives, the slightly lower recall suggests that a small number of true meningioma cases were not identified by the model. However, the overall F1-score of 0.98 reflects a strong balance between precision and recall. For the “no tumor” class, the precision, recall, and F1-score were all 1.00. This perfect score indicates that the model flawlessly identified the absence of tumors without any false positives or false negatives. The “pituitary tumor” class had a precision of 0.98, a recall of 0.99, and an F1-score of 0.99. This demonstrates the model’s robustness in detecting pituitary tumors with minimal false positives and a high rate of correctly identified true cases. Overall, the analysis of precision, recall, and F1-scores across different classes shows that the model performs exceptionally well in distinguishing between various types of brain tumors and the absence of tumors. The high scores across these metrics underscore the model’s reliability and effectiveness in medical imaging classification tasks.

**Fig 4:**
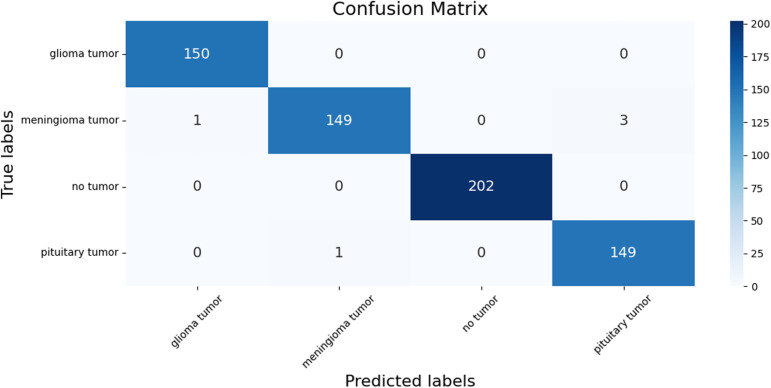
Confusion matrix of the brain tumor classification.

Moreover, a confusion matrix was computed to provide a detailed breakdown of the model’s classification performance across different brain tumor types. The confusion matrix was computed using the “confusion_matrix” function, and a heatmap of the confusion matrix was plotted, as shown in Fig 4.

Analyzing the confusion matrix reveals that the model demonstrates strong accuracy across all categories, with minor misclassifications primarily between meningioma tumors and pituitary tumors. Specifically, for glioma tumors, out of 150 cases, the model accurately predicted all 150, achieving a perfect classification accuracy of 100%. In the case of meningioma tumors, the model correctly identified 149 out of 153 cases, resulting in an accuracy of approximately 97.39%. However, there were some minor misclassifications: one case was misclassified as glioma, and three cases as pituitary tumors. These results suggest that while the model performs well, it occasionally struggles to distinguish between certain characteristics of meningioma and other tumor types, leading to a few misclassification errors.

For cases with no tumor, the model’s performance was exceptional, achieving a perfect classification accuracy of 100%. All 202 instances of no tumor were correctly identified, indicating the model’s proficiency in recognizing normal brain scans. In the classification of pituitary tumors, the model accurately predicted 149 out of 150 cases, resulting in an accuracy of approximately 99.33%. There was one misclassification where a pituitary tumor was incorrectly identified as a meningioma tumor. Overall, the confusion matrix highlights the model’s high accuracy and reliability in classifying brain tumor types.

Furthermore, the sensitivity, specificity, and accuracy for each class were also calculated and tabulated in Table 4.

**Table 4:** Sensitivity, specificity, and accuracy for each class.

	Sensitivity (%)	Specificity (%)	Accuracy (%)
**Glioma tumor**	100.00	99.80	100.00
**Meningioma tumor**	97.39	99.80	97.39
**No tumor**	100.00	100.00	100.00
**Pituitary tumor**	99.33	99.41	99.33
**Overall**	**99.18**	**99.75**	**99.24**

Based on Table 4, it is observed that the system exhibited remarkable performance, with sensitivities ranging from 97.39% to 100.00%, specificities from 99.34% to 100.00%, and accuracies from 97.39% to 100.00%. Specifically, the sensitivity for glioma tumors was 100%, and the specificity was 99.80%, indicating the model’s excellent ability to correctly identify glioma tumors without falsely labeling other tumors as glioma. For meningioma tumors, the sensitivity was 97.39% and the specificity was 99.80%, reflecting the model’s strong performance in identifying meningioma tumors while minimizing false positives. For no tumor cases, both sensitivity and specificity were 100%, showcasing the model’s flawless detection of the absence of tumors. For pituitary tumors, the model achieved a sensitivity of 99.33% and a specificity of 99.41%, demonstrating its robustness in detecting this specific tumor type.

As a result, the overall sensitivity of the model was 99.18%, and the overall specificity was 99.75%, highlighting the model’s high reliability in distinguishing between different classes. The per-class accuracy rates were 100% for glioma tumors, 97.39% for meningioma tumors, 100% for no tumor, and 99.33% for pituitary tumors. The model achieved an overall classification accuracy of 99.24%, indicating excellent performance on the test dataset.

In addition, the brain tumor classification system was also manually tested, verifying its accuracy. In order to predict and classify the brain tumor MRI images into four target classes of brain conditions (normal, glioma, meningioma, and pituitary), MRI images were randomly chosen and loaded into the system. For example, when a random brain MRI image was chosen and loaded from the pituitary folder, the word “pituitary” was printed out together with the image loaded, which indicated that correct outputs were obtained. The process of manual testing was repeated by loading MRI images from different classes. As a result, the designed system had correct predictions on randomly chosen MRI images.

### Web application

A web application was developed using the Dash framework to create a user-friendly GUI that allows users to upload MRI brain images and obtain predictions regarding the presence and type of tumor. The GUI was designed using HTML and Dash components, incorporating features such as an image upload button, an image display area, and a section to present prediction results and interesting facts about the predicted tumor type.

**Fig 5:**
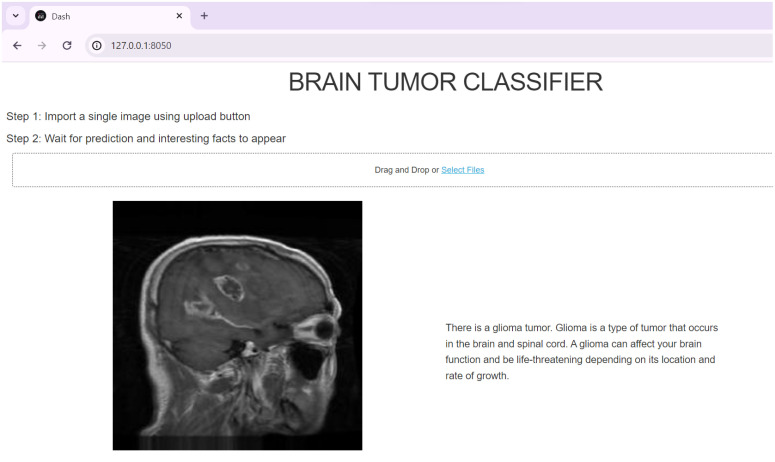
Web application output for uploaded glioma MRI image.

The GUI interacted with the classification model by processing the uploaded image, passing it through the model, and displaying the results. It enhanced the user experience by guiding users through the image upload process and providing informative details about the predicted tumor type based on the model’s prediction. As a result, the web application successfully performed accurate predictions of tumor types on randomly selected and uploaded MRI images. Fig 5 displays the output when a glioma MRI image was selected and uploaded, showcasing the GUI’s functionality and the corresponding prediction.

Overall, the proposed approach demonstrated effective brain tumor classification performance, as evidenced by the statistical analyses, performance metrics, and visualizations presented.

## Discussion

The implementation of our DL-based brain tumor classification model achieved an overall accuracy of 99.24%, significantly surpassing the results of previous studies, particularly those by M. Abu et al. [14] and Pillai et al. [15]. Both of these studies utilized variations of the pretrained VGG-16-based CNN architecture, similar to our approach, but their models yielded lower accuracies, with M. Abu et al. achieving 96% and Pillai et al. achieving 91.58%. The notable differences in accuracy between these studies and the current research can be attributed to several enhancements and modifications in our methodology.

Both M. Abu et al. and Pillai et al. employed transfer learning using the VGG16 architecture, yet their approaches differed notably in their execution. M. Abu et al. utilized a modest dataset of 251 MRI scans, applying minimal data augmentation techniques and adding four fine-tuned layers to the VGG16 model. While their approach achieved an accuracy of 96%, the limited dataset size may have hindered the model’s ability to generalize effectively, potentially leading to overfitting. Similarly, Pillai et al. achieved an accuracy of 91.58% with their methodology but did not leverage extensive data augmentation or optimization techniques, resulting in lower performance compared to the current study.

In contrast, the proposed model achieved an overall accuracy of 99.24%, surpassing the aforementioned studies significantly. A critical factor contributing to this success is the substantial increase in dataset size. While M. Abu et al. and Pillai et al. focused on binary classification (tumor versus non-tumor images) with relatively small datasets of 253 and 251 brain MRI images, respectively, our work expanded the classification task to four categories: glioma, meningioma, pituitary tumors, and normal brain scans. This was achieved using a significantly larger dataset of 17,136 MRI images, providing the model with diverse examples to learn from. The comprehensive dataset played a crucial role in training a robust and accurate model, enabling it to capture the complexities and variabilities associated with different brain tumors more effectively.

The architectural modifications implemented in our model also play a pivotal role in its superior performance. By incorporating additional layers and optimizing the parameters of the VGG16 architecture, our model effectively enhances its feature extraction capabilities. The introduction of a flatten layer reshaped feature maps into a one-dimensional vector, facilitating efficient processing by subsequent dense layers. Furthermore, the addition of dense layers with the ReLU activation function introduced nonlinearity and enabled the model to learn complex patterns and abstract representations. By incorporating 256 neurons in this layer, the model was able to capture a wide range of features, enhancing its discrimination between different tumor types. The final dense layer, consisting of 4 neurons and employing softmax activation, played a vital role in generating meaningful class probabilities. This activation function produced confidence scores for each tumor class, enabling accurate identification and classification. In summary, the choice of additional layers and optimal parameters in the modified VGG-16 model greatly contributed to the observed improvement in performance. The flatten layer facilitated efficient processing, the dense layers with ReLU activation enhanced the model’s ability to learn complex patterns, and the final dense layer with softmax activation provided meaningful class probabilities. These modifications collectively enhanced the model’s capacity to extract relevant features and classify brain tumors accurately.

Besides, our implementation of a two-stage training approach stands out as a significant enhancement over previous methodologies. This strategy allowed for effective fine-tuning of the pretrained VGG-16 layers, which enhanced the model’s feature extraction capabilities. Initially, the newly added layers were trained while keeping the pretrained layers of the VGG-16 model frozen. This allowed the model to adapt the new classification layers to the brain tumor dataset. In the subsequent stage, some of the pretrained layers were unfrozen and fine-tuned with a lower learning rate. This strategic approach enhanced the model’s feature extraction capabilities, allowing it to adjust the pretrained weights to align more closely with the specific task of brain tumor classification.

The extensive data augmentation strategy employed in our study further distinguishes it from existing methodologies. By introducing slight modifications to the original images through techniques such as rescaling, shearing, zooming, and horizontal flipping, additional training samples with increased variations were created. This augmentation increased the diversity of the dataset, allowing the model to learn from a broader range of examples and improving its ability to generalize to unseen data. The augmented dataset with increased variability helped the model to better capture the complexities and variabilities associated with brain tumors, leading to improved performance. In contrast, both M. Abu et al. and Pillai et al. relied on limited data augmentation strategies, which likely contributed to their lower accuracy rates.

Besides, normalizing pixel values to a range of 0–1 was a common practice to ensure consistency in input data. This is because keeping pixel values at their original range of 0–255 would make the training process less stable, slower, and potentially less effective. Thus, this step facilitated stable and faster convergence during training by removing biases introduced by varying pixel scales. It helped to achieve smoother training curves and more consistent accuracy, contributing to a more robust learning process. Rescaling as part of normalization indeed played a crucial role in training convergence. Models trained with normalized data generally exhibit better performance metrics, including higher accuracy and lower loss, as normalization ensures that input data is within a manageable range for the model.

The impact of normalization and rescaling extends beyond the specific CNN model used in this study and is relevant to a variety of DL algorithms. In CNNs, normalization is essential for standardizing input data, which helps to stabilize and expedite the training process. Similarly, fully connected neural networks also benefit from normalization, as it prevents issues like exploding or vanishing gradients by keeping the inputs within a suitable range. The preprocessing steps of data augmentation, normalization, and rescaling were crucial in improving the model’s performance and generalization ability. These techniques not only enhanced the accuracy and robustness of the brain tumor classification model used in this study but also have broad applicability and benefits across various DL algorithms and architectures. This holistic approach to preprocessing ensures that models can learn effectively from the training data, leading to more reliable and accurate predictions.

The accuracy results reported for different tumor classes further validate the effectiveness of the implemented approach. The model achieved high accuracy across various tumor classes, with the “meningioma tumor” and “pituitary tumor” classes achieving accuracies of 97.39% and 99.33%, respectively. Moreover, both the “no tumor” and “glioma tumor” classes achieved a perfect accuracy of 100%. These results demonstrated the model’s strong performance in accurately classifying different types of brain tumors.

The impact of increasing the number of epochs on model accuracy was also explored in this study by adjusting the parameters in two training stages. Increasing the number of epochs in both two training stages resulted in an increase in model accuracy. This suggests that the model continued to benefit from additional training epochs and was able to further improve its performance. However, it is important to monitor the validation accuracy during training to prevent overfitting. The model’s validation accuracy and loss did not exhibit significant changes beyond the initial 50 epochs, likely due to the very small learning rate (0.0001) applied during the training after the first 50 epochs. The small learning rate caused the model to learn slowly, requiring more time to achieve better validation accuracy and loss. Although there was an overall improvement in loss and accuracy, the validation accuracy and loss did not demonstrate substantial changes beyond the initial epochs.

Despite the success and promising results of this study, it is important to acknowledge certain limitations. Further validation and testing on larger and more diverse datasets are essential to assess the generalizability and real-world performance of the system. The dataset used in this study, although large and diverse, did not fully represent the entire spectrum of brain tumors. Expanding the dataset size and diversity could provide a more comprehensive evaluation of the system’s capabilities and ensure its applicability to a broader range of clinical scenarios. The system focused on the classification of four specific brain tumor types, and future research may explore the inclusion of a broader range of brain tumor categories to enhance its clinical utility. Given the heterogeneity of brain tumors, the incorporation of more tumor types in the training dataset could contribute to improved performance and increase the system’s reliability in diagnosing various brain tumors.

To further enhance the system’s performance, future research may explore the use of alternative network architectures or hybrid models, comparing their efficacy in brain tumor classification. Comparative studies involving various network architectures may provide valuable insights into the most effective approach for accurate brain tumor classification.

In conclusion, the proposed DL-based brain tumor classification model demonstrates a marked improvement in accuracy over existing methods. The implemented DL-based brain tumor classification approach, incorporating a modified VGG-16 model with a refined network configuration, a two-stage training approach, data augmentation techniques, and a large and diverse dataset, has achieved remarkable accuracy and holds significant promise for assisting medical professionals in the timely and accurate diagnosis of brain tumors. These methodological advancements not only underscore the potential of DL in the medical field but also pave the way for the development of more effective diagnostic tools for brain tumors.

## Conclusion

This study successfully developed a brain tumor classification system leveraging Python and the VGG16 network as a pretrained model, achieving an impressive overall classification accuracy of 99.24% on a dataset comprising glioma, meningioma, pituitary tumors, and normal MRI images. The use of a CNN with the pretrained VGG16 architecture demonstrated accurate predictions on randomly selected brain MRI images, underscoring its potential to assist clinicians in the early screening and diagnosis of brain tumors.

The contributions of this research are significant: the incorporation of a larger and more comprehensive dataset, created by combining data from FIGSHARE, SARTAJ, and BR35 in a balanced ratio, enhances the model’s robustness and reliability. This study advances the classification of brain tumors from a binary to a four-class approach, providing detailed diagnostic information crucial for personalized treatment planning. In addition, the research validates an enhanced DL model utilizing advanced techniques such as data augmentation and fine-tuning, ensuring high accuracy and generalizability. A user-friendly web application has also been developed based on the validated model, facilitating real-time predictions and easy integration into existing healthcare systems.

Looking ahead, future research should focus on expanding the dataset to include a broader range of tumor types, particularly rare or less common brain tumors, to improve the model’s generalizability. Exploring alternative DL architectures or hybrid models could yield insights into more effective classification techniques. Moreover, conducting clinical trials to validate the model in real healthcare settings is essential for assessing its practical applicability. Lastly, integrating other imaging modalities, such as PET or CT scans, may enhance diagnostic accuracy and provide a more comprehensive analysis of brain tumors.
